# Pre-clinical Evaluation of Shilajit in Cancer: A Systematic Review

**DOI:** 10.7759/cureus.101736

**Published:** 2026-01-17

**Authors:** Sushant S Das, M Ramkumar, Harsimranjit Singh, Shalika Sharma

**Affiliations:** 1 Department of Anatomy, All India Institute of Medical Sciences Vijaypur, Jammu, Jammu, IND

**Keywords:** anti-cancer, cancer, mechanism, preclinical, shilajit, systematic review

## Abstract

Shilajit, an Ayurvedic herbo-mineral preparation containing fulvic acids and humic substances, has been studied for its anti-cancer potential; however, a precise summary of experimental evidence is not available. The purpose of this systematic review and qualitative synthesis was to analyse the preclinical and clinical evidence on the anticancer potential, mechanisms, and translational prospects of Shilajit. PubMed, Ovid Discovery, and Google Scholar were searched systematically, and only nine preclinical studies met predetermined inclusion and exclusion criteria. No clinical studies were eligible. Data on cytotoxicity, mechanisms of action, and selectivity were extracted by two independent reviewers. These included studies (eight in vitro and one in vivo osteosarcoma model) analysed the effect of Shilajit on various types of cancer. Despite differences in methodologies, all studies reported dose-dependent cytotoxicity with IC₅₀ values ranging from 31 to 1103 µg/mL for various cancer types, depending on the type of cancer and form of Shilajit preparation. Selectivity against cancer cells was observed as compared to normal cell lines. Molecularly, Shilajit mediated apoptosis through the generation of reactive oxygen species and inhibition of NF-κ B (Nuclear Factor kappa-light-chain-enhancer of activated B cells)/IKK (I-kappa-B kinase) signaling, as well as suppression of epithelial-mesenchymal transition and cell cycle arrest. In vivo findings suggested chemopotentiation and organ protection when administered together with standard therapy. Thus, Shilajit shows multitargeted anticancer activities in various experimental cancer models by several mechanisms. This evidence is preclinical, and standardization issues restrict clinical translation. The results support continued investigation through rigorous, blinded studies, recognising that no clinical trials currently exist and that any potential clinical translation remains speculative at this stage.

## Introduction and background

Shilajit is a blackish‑brown herbo‑mineral exudate long used in Ayurveda as a rejuvenating remedy (*rasayana*) and broad‑spectrum tonic, traditionally credited with benefits ranging from enhanced vitality to treatment of chronic diseases [1-3]. These historical claims have only partially been explored in modern biomedical research.

Chemically, it is rich in humic substances-fulvic and humic acids, along with dibenzo‑α‑pyrones, minerals, and trace elements, which underpin experimentally observed antioxidant, anti‑inflammatory, immunomodulatory, anti-mutagenic, and antitumor activities in preclinical models rather than clinically established effects in humans [2-4].

Over the past decade, rising interest in natural products for cancer prevention and adjunctive therapy has driven a wave of preclinical research on Shilajit within the broader field of integrative oncology, where evidence-informed use requires a clear distinction between traditional use, laboratory findings, and rigorously tested clinical interventions. In vitro studies across multiple human cancer cell lines (breast, lung, liver, ovarian, cervical, colorectal, bladder, and oral cancers) consistently show dose‑ and time‑dependent cytotoxicity, induction of apoptosis, cell‑cycle arrest, and inhibition of migration and invasion, often with relative sparing of normal cells [5-10].

Proposed mechanisms include modulation of pro‑ and anti‑apoptotic genes, generation of reactive oxygen species, suppression of NF-κ B (nuclear factor kappa-light-chain-enhancer of activated B cells)/IKK (I-kappa-B kinase) and other oncogenic pathways, and interference with urokinase‑type plasminogen activator and epithelial‑mesenchymal transition signaling [5-10]. Animal data suggest Shilajit may also attenuate chemotherapy‑associated organ toxicity and potentiate antitumor effects in osteosarcoma models [11]. However, as per our search, no randomized clinical trials have yet evaluated Shilajit as an anticancer agent in humans, and concerns remain about dose, standardization, and potential interactions, especially its ability in some settings to protect tumor cells or blunt chemotherapeutic cytotoxicity [1,2,6,11]. Against this background of promising but fragmented evidence, an evidence‑based systematic review is warranted to critically appraise and synthesize the available data on Shilajit in cancer, clarify its mechanistic plausibility, and identify key gaps that must be addressed before clinical translation.

## Review

Materials and methods

This systematic review followed the Preferred Reporting Items for Systematic Reviews and Meta-Analyses (PRISMA) guidelines [12]. To decrease the risk of bias in the review, the authors prepared a systematic review protocol and got it prospectively registered with PROSPERO (International Prospective Register of Systematic Reviews) at the Centre for Reviews and Dissemination, the University of York (registration ID: CRD420251128736) before the data extraction. The review protocol can be accessed from https://www.crd.york.ac.uk/PROSPERO/view/CRD420251128736.

Search Strategy

A preliminary online search was done of published articles in PubMed, Ovid Discovery, and Google Scholar in August 2025 using the following Medical Subject Headings (MeSH) terms: (“Shilajit” OR “Asphaltum punjabianum” OR “Mineral pitch”) AND “Cancer”. Also, a manual search of the reference lists of the included studies was done to find other relevant papers.

Eligibility Criteria

Studies were considered eligible for inclusion if they provided preclinical or clinical evidence evaluating the anticancer effects of Shilajit. Preclinical studies were eligible if they involved experimental investigations conducted in human cancer cell lines or animal models of any cancer type in which Shilajit or its bioactive constituents were assessed. Also, clinical studies were eligible if they involved human participants of any age, sex, or ethnicity with a confirmed diagnosis of cancer and evaluated Shilajit as an intervention, either as monotherapy or as an adjunct to standard anticancer treatments. No restrictions were imposed on the year or language of publication.

Studies were excluded if the primary condition under investigation was not cancer or if Shilajit was not clearly identifiable as the principal intervention, including studies where it was administered as part of a multi-component formulation without the ability to isolate its specific effects. Moreover, the exclusion criteria included non-peer-reviewed literature, abstract-only articles, case reports, case series, proceedings of the conference, letters to the editor, commentaries, short communications, and publications lacking the full text.

Study Selection and Screening

Two independent reviewers screened the titles and abstracts of articles to seek the relevant articles based on the criteria of eligibility. The full articles of eligible studies were then obtained to determine their appropriateness for the review. Any differences between the two reviewers were clarified by a third reviewer.

Data Extraction

Data were extracted independently by two authors using a data extraction form made using Microsoft Excel (Microsoft Corporation, Redmond, Washington, United States). From each included study, the following information was collected: (i) General study characteristics: author names, year of publication, country, study design (in vitro, in vivo animal, clinical trial, observational study, etc.); (ii) Population/model details: for in vitro studies: cell line type and origin; for animal studies: species, strain, sex, age, and sample size, tumor model (induced/spontaneous; method of induction); for clinical studies: number of participants and demographic characteristics (age, sex, cancer type and stage), inclusion and exclusion criteria; (iii) Intervention details (Shilajit): type and source of Shilajit or its bioactive component, preparation/formulation (e.g., purified extract, standardized product, compound), dose, route of administration, frequency, and duration, co-interventions (e.g., chemotherapy, radiotherapy, other agents); (iv) Comparator(s): type of control: vehicle, no treatment, placebo, standard therapy, or other active comparator, dose and regimen of comparator; (v) Outcomes: primary anticancer outcomes (e.g., cell viability, apoptosis, cell cycle arrest, tumor volume, tumor weight, survival); secondary outcomes (e.g., oxidative stress markers, inflammatory markers, immune parameters, quality of life, adverse events), time points of outcome measurement, effect estimates (e.g., mean/standard deviation, risk ratios, hazard ratios) and statistical significance; (iv) Other relevant information: mechanistic findings (e.g., signaling pathways, molecular targets), authors’ main conclusions and limitations reported by the authors.

Any disagreements between reviewers were settled by discussion or consultation with a third reviewer. The study's authors were contacted if any data were missing or ambiguous.

Data Quality

The extracted data's overall quality was assessed based on its consistency, completeness, and bias risk. For every study that was included, we evaluated the following important methodological aspects: study design, sample size, cell lines, cancer type, intervention details, comparators, and outcomes. When essential information was missing or unclear, we attempted to clarify this by consulting supplementary materials or contacting authors where feasible. Studies with insufficient reporting that precluded reliable effect estimation were documented and retained only for qualitative synthesis.

Risk of Bias

Two independent reviewers assessed the risk of bias of the included studies using design‑specific tools, with differences resolved by discussion or a third reviewer. The eight in vitro studies [7-9,13-17] were assessed using the ToxRTool for in vitro studies [18]. This tool analysed risk of bias based on the following criteria: test substance identification, test substance characterization, study design description, study results documentation, and plausibility of study design and data. Each study was rated as reliable without restrictions, reliable with restrictions, not reliable, or not assignable based on the scoring.

The single in vivo study [11] was evaluated using the Systematic Review Center for Laboratory Animal Experimentation (SYRCLE) tool for animal studies [19], which was adapted from the Cochrane risk of bias instrument for use in animal experiments. This tool addresses selection, performance, detection, attrition, reporting, and other biases through 10 signaling‑question-based domains. The findings were classified as low, high, or unclear risk based on the reported methods.

Results

Literature Retrieval

**Figure 1:**
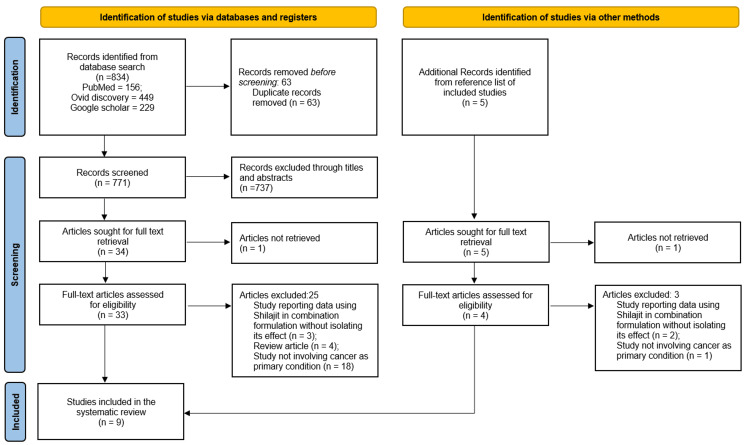
PRISMA flow diagram illustrating study selection and inclusion process PRISMA: Preferred Reporting Items for Systematic Reviews and Meta-Analyses

A total of 834 articles were selected from PubMed, Ovid Discovery, and Google Scholar. After duplication removal and initial screening, 34 articles remained. Upon full-text review, 26 studies were excluded due to significant methodological errors or insignificance to the current research. One more article of possible interest was found by searching the reference lists of the finally included articles. Ultimately, the review included nine articles that met all the inclusion and exclusion criteria (Figure 1).

Study Characteristics

The study characteristics of the nine included studies are summarised in Table 1. All the studies were preclinical experimental studies, comprising eight in vitro cancer studies [7-9,13-17], including two with combined in vitro cytotoxicity assays with in vivo experiments that were not cancer-specific [13,17]. One study employed an in vivo cancer model [11]. No randomized controlled clinical trials or clinical human studies evaluating Shilajit as a primary anticancer therapy were identified. The included studies were published between 2008 and 2025 and originated predominantly from Asia and the Middle East.

**Table 1:** Characteristics of included studies evaluating anticancer effects of Shilajit ABTS: 2,2'-azino-bis(3-ethylbenzothiazoline-6-sulfonic acid), ALP, alkaline phosphatase: ALT, alanine aminotransferase, AO/EtBr: acridine orange/ethidium bromide, As: Arsenic, AST: aspartate aminotransferase, CMF: cyclophosphamide/methotrexate/5-fluorouracil, DAPI: 4',6-diamidino-2-phenylindole, DMSO: dimethyl sulfoxide, DPPH: 2,2-diphenyl-1-picrylhydrazyl, ED₅₀: median effective dose, EMT: epithelial-mesenchymal transition, ER+: estrogen receptor-positive, H&E: hematoxylin and eosin, HBSS: Hank's balanced salt solution, HDS: high-dose Shilajit (250 mg/kg), Hg: Mercury, hGF: human gingival fibroblasts, IC₅₀: half-maximal inhibitory concentration, ICP-MS: inductively coupled plasma-mass spectrometry, IKK: inhibitor of κB kinase, LCMS: liquid chromatography-mass spectrometry, LDS: low-dose Shilajit (150 mg/kg), miR: microRNA, MTT: 3-(4,5-dimethylthiazol-2-yl)-2,5-diphenyltetrazolium bromide, NAC: N-acetyl cysteine, NF-κB: nuclear factor-κB, NO: nitric oxide, OS: osteosarcoma, PARP: poly (ADP-ribose) polymerase, Pb: Lead, qRT-PCR: quantitative reverse transcription polymerase chain reaction, ROS: reactive oxygen species, TGF-β: transforming growth factor-beta, TP: total protein, TUNEL: terminal deoxynucleotidyl transferase dUTP nick end labeling, uPA: urokinase-type plasminogen activator, uPAR: urokinase plasminogen activator receptor.

S.No.	Study (Author(s), Year)	Country	Design	Population/Model	Intervention	Comparator	Primary Outcomes	Key Mechanisms	Main Conclusions
1	Phaechamud et al. 2008 [17]	Thailand	In vitro (also includes in vivo, which was not cancer-specific)	Human cell lines- MRC-5 (normal lung fibroblast), A549 (lung cancer), HepG2 (liver cancer), MDA-MB-231 (breast cancer), SW-620 (colorectal adenocarcinoma), SKOV-3 (ovarian cancer), HeLa (cervical cancer)	Mineral pitch (aqueous/DMSO extract), concentrations variable	Vehicle/ untreated control	Cell viability (MTT), ED₅₀, antimicrobial activity, antifungal activity, antioxidant (ABTS), heavy metal analysis (ICP-MS)	Broad-spectrum bioactivity, antioxidant activity, ROS generation (inferred)	ED₅₀ (24 h): A549 (89 µg/mL), HepG2 (96 µg/mL), MDA-MB-231 (225 µg/mL), SKOV-3 (286 µg/mL); HeLa most sensitive (31.5% viability at 100 µg/mL); MRC-5 normal cells (24.8% inhibition); variable potency; high heavy metal content (As, Hg, Pb)
2	Pant et al. 2016 [15]	India	In vitro	Human cell lines- Huh-7 (Hepatocellular carcinoma)	Mineral pitch (DMSO/aqueous extract, western Nepal), 10-500 µg/mL, 24-48 h	Vehicle control (DMSO)	Cell viability (MTT), apoptosis (TUNEL), ROS/NO production, IC₅₀	ROS-mediated apoptosis, caspase-3 activation, PARP cleavage, miRNA modulation (↑miR-22, ↓miR-21), c-Myc suppression	IC₅₀ (24-48 h): 100-500 µg/mL; ROS scavenger (NAC) reversed apoptosis; miRNA-22 overexpression inhibited proliferation 4.9-fold; strong mechanistic evidence
3	Jafari et al. 2019 [13]	Iran	In vitro (also includes in vivo, which was not cancer-specific)	Human cell lines- MCF-7 (breast cancer), A549 (lung cancer)	Native Shilajit (aqueous extract, Bahr Aseman mountains), 10-500 µg/mL, 24-72 h	Untreated control	Cell viability (MTT), IC₅₀, cytotoxicity, antioxidant (DPPH)	Moderate ROS generation, free radical scavenging	IC₅₀ (24 h): MCF-7 (727.5 µg/mL), A549 (1103 µg/mL); selective cytotoxicity; antioxidant at high concentrations
4	Rahmani Barouji et al. 2020 [16]	Iran	In vitro	Human cell lines- MCF-7(breast cancer), MDA-MB-231 (breast cancer), MCF-10A (normal mammary epithelial)	Iranian Mummy (pharmaceutical grade, Tuba company), 10-100 µg/mL, 24-48 h	Untreated control	Cell viability (MTT), IC₅₀, apoptosis (Annexin-V/PI, DAPI), EMT gene expression (qRT-PCR)	EMT inhibition: ↓TGF-β1, ↓TGF-βR1, ↓TWIST1, ↓NOTCH1, ↓CTNNB1 (β-catenin), ↓SRC, ↓vimentin; ↑E-cadherin	IC₅₀ (24 h): MCF-7 (40 µg/mL), MDA-MB-231 (31.3 µg/mL), MCF-10A (89.7 µg/mL); 67.3% apoptosis (MDA-MB-231), 59.8% (MCF-7); EMT reversal; selectivity index 2.2-2.9
5	Kloskowski et al. 2021 [9]	Poland	In vitro	Human cell lines- T24, 5637 (bladder cancer), SV-HUC1 (normal urothelium)	Mumio (Shilajit, Poland source), 10-100 µg/mL, 24-72 h	Vehicle control	Cell viability (MTT), IC₅₀, apoptosis (Annexin-V/PI), cell cycle (flow cytometry)	Cell cycle arrest (G0/G1, S phase), caspase-dependent apoptosis, selective cancer toxicity	IC₅₀ (24-72 h): 30-50 µg/mL (T24, 5637); minimal toxicity to SV-HUC1 normal cells; G0/G1 arrest followed by apoptosis
6	AlShubaily 2022 [14]	Saudi Arabia	In vitro	Human cell lines- MCF-7 (breast cancer), T-47D (breast cancer), HepG2 (liver)	Shilajit extract (LCMS-profiled, 17 compounds identified), concentration range variable	Vehicle/ untreated control	Cytotoxicity, IC₅₀, antimicrobial, antifungal activity	Chemical profiling (LCMS), broad-spectrum bioactivity	Potent cytotoxicity in HepG2 ( IC₅₀= 19 µg/mL); weaker activity in MCF-7 and T-47D; bioactives identified (fulvic acid, gallic acid, ferulic acid, dihydrodibenzo-pyrones, pregnane)
7	Jambi and Abdulaziz Alshubaily [11]	Saudi Arabia	In vivo animal	Male Sprague-Dawley rats (N=35, 5 groups, n=7 each), osteosarcoma model (4T1 cells, 1.0×10⁴ cells injected into femoral medullary cavity), ~220g initial weight	Shilajit extract (Natural Spirit Trading Est, Riyadh; dissolved in HBSS, filtered 0.2 mm), Low dose 150 mg/kg, High dose 250 mg/kg, oral (route inferred), duration not reported	Control (saline), OS alone, OS + CMF chemotherapy (cyclophosphamide 10 mg/kg, methotrexate 1 mg/kg, 5-FU 10 mg/kg)	Body weight, serum liver markers (ALT, AST, ALP, bilirubin, albumin, TP), kidney markers (creatinine, urea, uric acid), liver & kidney histopathology (H&E)	Chemopotentiation, hepatoprotection, nephroprotection, functional biomarker normalization, histological restoration	HDS+CMF reversed OS-induced dysfunction: Liver ALT 49.57±1.71 vs. 99.85±5.87 U/L (OS alone); Kidney creatinine 4.98±0.106 vs. 7.41±0.22 mg/dL (OS alone); HDS superior to LDS (p<0.05); near-normal tissue architecture achieved
8	Kordestani et al. 2024 [8]	Iran	In vitro	Human cell lines- MCF-7 (breast cancer)	Shilajit extract (Iran source), 100-800 µg/mL, 24-72 h	Untreated control	Cell viability (MTT), IC₅₀, apoptosis (Annexin-V/PI flow cytometry), gene expression (qRT-PCR)	IKK/NF-κB pathway inhibition: ↓IKKα, ↓IKKβ, ↓p50 (0.31-fold), ↓RelB (0.22-fold)	IC₅₀: 280 µg/mL (72 h); dose- and time-dependent cytotoxicity; 43% apoptosis induction at IC₅₀; NF-κB suppression confirmed
9	Alqarni et al. 2025 [7]	Saudi Arabia	In vitro	Human cell lines- KB-1 (oral squamous cell carcinoma), hGF (normal gingival fibroblasts)	Shilajit (Lajit Gold, Himalayan source), 0.25-1.5 mg/mL (cancer cells), 0.5-6 mg/mL (normal cells), 24-48 h	Untreated control/ vehicle	Cell viability (MTT), IC₅₀, apoptosis (Annexin-V/PI, AO/EtBr), migration (scratch assay), gene expression (qRT-PCR)	uPA/uPAR suppression, chemokine signaling inhibition (↓CXCL8, ↓CXCR2), proapoptotic gene upregulation (↑p53, ↑Bax, ↑caspase-3), ↓Bcl-2	IC₅₀: KB-1 1.049±0.122 mg/mL (24 h), 0.965±0.026 mg/mL (48 h); hGF: 4-5 mg/mL; 32.37% apoptosis; migration significantly inhibited; selectivity for cancer cells

The cancer types investigated were varied and included breast cancer, oral squamous cell carcinoma, hepatocellular carcinoma, urinary bladder cancer, lung cancer, ovarian cancer, and osteosarcoma. In vitro studies [7-9,13-17] utilized a variety of established human cancer cell lines, such as MCF-7 [8,13,14,16] and MDA-MB-231 (breast cancer) [16,17], KB-1 (oral cancer) [7], HeLa (cervical cancer) [17], HepG2 [14,17] and Huh-7 (liver cancer) [15], T24 and 5637 (bladder cancer) [9], A549 (lung cancer) [13], and SKOV-3 (ovarian cancer) [17], with several studies including normal cell lines [7,9,16,17] as controls to assess selectivity. One in vivo study [11] employed an osteosarcoma rat model to evaluate the adjunctive and organ-protective effects of Shilajit in combination with standard chemotherapy.

Across studies, Shilajit was administered in various aqueous [13,15,17], dimethyl sulfoxide (DMSO) [15,17], or crude extract forms [7-9,16], with concentrations ranging from low micromolar to milligram per milliliter levels in vitro, and dose-dependent regimens in animal models. Primary outcomes assessed included cell viability [7-9,13,15-17], apoptosis [7-9,15,16], cell cycle arrest [9], oxidative stress markers [13,15,17], migration and invasion assays [7,16], gene and protein expression [7,15,16], and histopathological evaluation [11] in animal tissues. Mechanistic outcomes frequently focused on reactive oxygen species generation, inflammatory and metastatic signaling pathways, epithelial-mesenchymal transition (EMT), microRNA (miRNA) modulation, and NF-κ B pathway inhibition. Despite methodological heterogeneity, all included studies consistently reported anticancer or cytoprotective effects attributable to Shilajit or its bioactive constituents. Also, a meta-analysis was not feasible due to the heterogeneity and insufficient comparable data.

Risk of Bias Assessment

In vitro studies: The summarised ToxRTool risk of bias results of the eight in vitro studies are presented in Table 2. Initial numerical scoring indicated higher reliability categories; however, applying ToxRTool's criterion of checking red scores for category revision, all eight studies [7-9,13-17] were reclassified as reliability Category 3 (not reliable) due to the absence of positive control substances. Despite this limitation, all in vitro studies were retained for analysis as they provided adequate test substance identification, dose-response characterization, and endpoint documentation essential for hazard identification. This approach aligns with established guidance permitting inclusion of Category 3 studies in weight-of-evidence assessments when methodological gaps do not compromise core findings [20].

**Table 2:** ToxRTool reliability assessment of the included in vitro studies

S.No.	Study (Author(s), Year)	Test substance	Test system	Key methodological limitations	Overall ToxRTool category
1	Phaechamud et al., 2008 [17]	Mineral pitch (Shilajit) extract	MRC-5 (normal lung fibroblast), A549 (lung cancer), HepG2 (liver cancer), MDA-MB-231 (breast cancer), SW-620 (colorectal adenocarcinoma), SKOV-3 (ovarian cancer), HeLa (cervical cancer)	No purity data; limited physicochemical characterization; no information on cell line source/authentication; incomplete culture conditions; no explicit replication; no statistical methods reported; no positive control	Category 3 (not reliable; numerical score category 2 but downgraded due to red-criterion failures)
2	Pant et al., 2016 [15]	Mineral pitch (Shilajit) extract	Huh-7 (Hepatocellular carcinoma)	No information on cell line source/authentication; no positive control	Category 3 (not reliable; numerical score category 1 but downgraded due to red-criterion failures)
3	Jafari et al., 2019 [13]	Native Shilajit aqueous extract	MCF-7 (breast cancer), A549 (lung cancer)	No purity data; no physicochemical characterization; no cell line source/authentication; replication not stated; limited statistical reporting; no positive control	Category 3 (not reliable; numerical score category 2 but downgraded due to red-criterion failures)
4	Rahmani Barouji et al., 2020 [16]	“Mummy” (Iranian Shilajit) extract	MCF-7(breast cancer), MDA-MB-231 (breast cancer), MCF-10A (normal mammary epithelial)	No cell line source/authentication; no physicochemical characterization; no positive control	Category 3 (not reliable; numerical score category 1 but downgraded due to red-criterion failures)
5	Kloskowski et al., 2021 [9]	Mumio (Shilajit) extract	T24, 5637 (bladder cancer), SV-HUC1 (normal urothelium)	No cell line source/authentication; no physicochemical characterization; no positive control	Category 3 (not reliable; numerical score category 1 but downgraded due to red-criterion failures)
6	AlShubaily, 2022 [14]	Shilajit extract	MCF-7 (breast cancer), T-47D (breast cancer), HepG2 (liver)	Limited information on test system sources; minimal culture conditions; replication not stated; limited statistics; no positive control	Category 3 (not reliable; numerical score category 2 but downgraded due to red-criterion failures)
7	Kordestani et al., 2024 [8]	Shilajit extract	MCF-7 (breast cancer)	No cell line source/authentication; no physicochemical characterization; no positive control	Category 3 (not reliable; numerical score category 1 but downgraded due to red-criterion failures)
8	Alqarni et al., 2025 [7]	Shilajit extract	KB-1 (oral squamous cell carcinoma), hGF (normal gingival fibroblasts)	No cell line source/authentication; no physicochemical characterization; no positive control	Category 3 (not reliable; numerical score category 1 but downgraded due to red-criterion failures)

**Figure 2:**
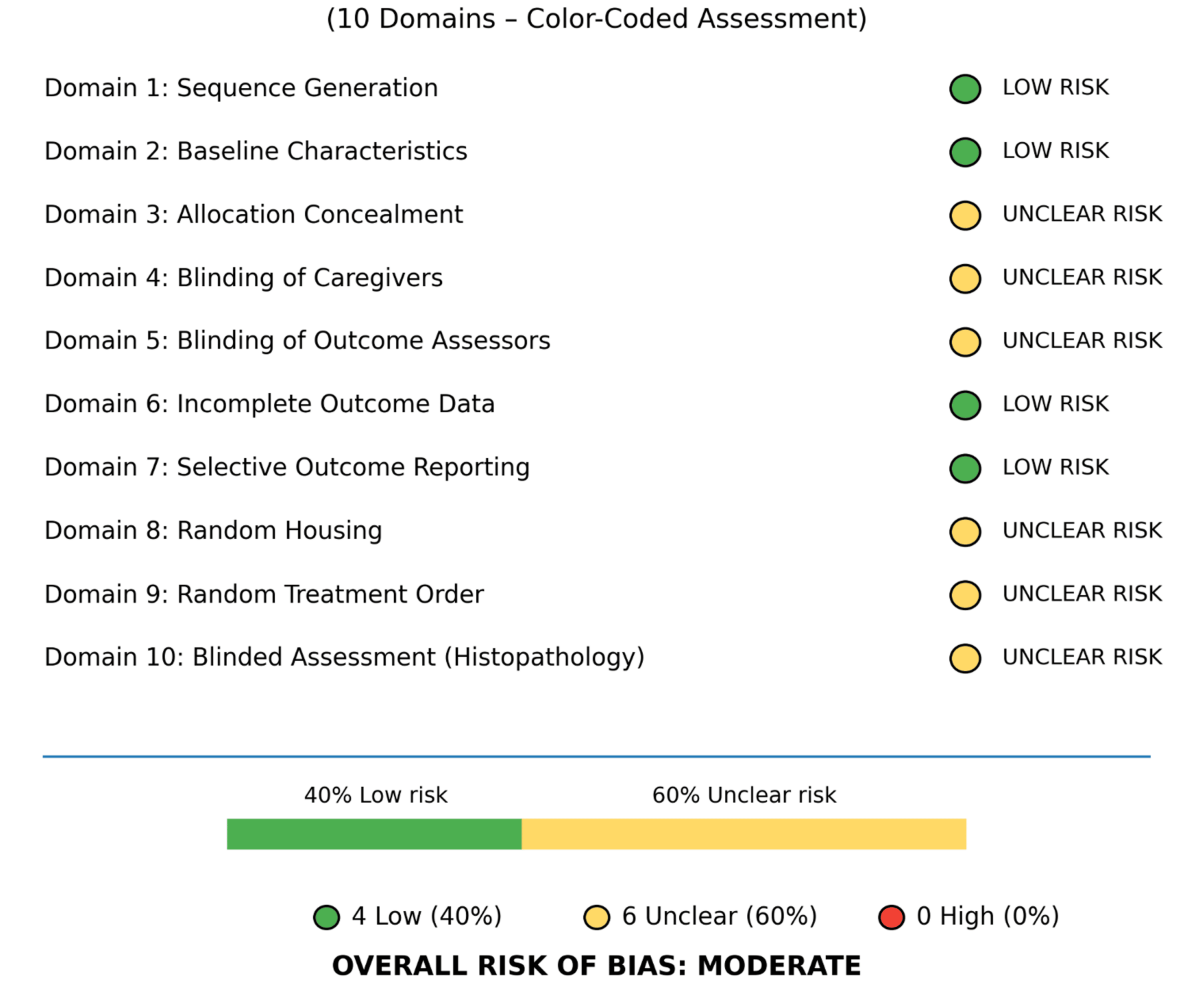
SYRCLE risk of bias assessment results of the in vivo study Reference: Jambi et al. 2022 [11] SYRCLE: Systematic Review Center for Laboratory Animal Experimentation

In vivo study: The risk of bias assessment according to SYRCLE is presented in Figure 2. Jambi et al.'s study demonstrated a moderate risk of bias [11]. Randomization and baseline characteristics were adequately reported; however, allocation concealment, random housing, and caregiver blinding were not described. The critical limitations included an unblinded histopathological assessment that could have biased the tissue grading findings. There were no incomplete outcome data and no selective reporting. Overall, 40% of the domains were low-risk and 60% unclear-risk, with primary concern for potential bias in the subjective evaluation of tissue.

Therefore, all nine studies were retained for synthesis following quality-adjusted interpretation protocols.

Discussion

This systematic review synthesizes the evidence from nine preclinical experimental studies assessing the anticancer potential of Shilajit and related preparations, such as mineral pitch or mumio, across various malignancies, experimental models, and mechanistic endpoints. Given heterogeneity in study design, Shilajit source, extraction method, and dosing, the collective findings nevertheless point to dose- and time-dependent anticancer activity consistently. The biological credibility of Shilajit as a promising anticancer agent or supplement to traditional therapy is strengthened by the convergence of these findings across various cancer types.

Cytotoxic Effects Across Cancer Types

Shilajit showed dose-dependent cytotoxicity against a variety of human cancer cell lines in a number of in vitro experiments [7-9,13-17]; the reported IC₅₀ (half maximal inhibitory concentration) values range from low tens of micrograms per millilitre to more than one milligram per millilitre. Such a broad range of values suggests variations in cancer cell biology, preparation of Shilajit, composition of the extract, length of exposure, and the assay technique.

In breast cancer models, significant variation in cytotoxic potency was seen. Jafari et al. reported IC₅₀ values of approximately 727.5 µg/mL for MCF-7 cells using an aqueous Shilajit extract, which represents moderate cytotoxicity [13]. On the contrary, Kordestani et al. revealed an IC₅₀ of 280 µg/mL in MCF-7 cells after 72 hours of incubation, indicating that the duration of exposure may play a role in augmenting antitumor activity [8]. More impressively, Rehmani Barouji et al., using Iranian “Mummy” or the purified Shilajit preparation, demonstrated a remarkably lower IC₅₀ of 31.3 µg/mL in the highly aggressive MDA-MB-231 breast cancer line and 40 µg/mL in MCF-7 cells with greater potency, and thus suggesting that the more aggressive, triple-negative-like phenotype may be especially sensitive to Shilajit [16]. More importantly, the same study reported that the IC₅₀ was 89.7 µg/mL in normal human mammary epithelial cells (MCF-10A), indicating a favourable selectivity index for malignant cells.

In hepatic cancer, Pant et al. assessed the activity of mineral pitch against Huh-7 hepatocellular carcinoma cells, reporting IC₅₀ values between 100-500 µg/mL, depending on concentration and the nature of the assay endpoint [15]. These findings place Shilajit in the range of moderate cytotoxicity for liver cancer.

In certain studies, lung cancer cells appeared to be relatively less sensitive. According to Jafari et al., A549 lung cancer cells had an IC₅₀ of 1103 µg/mL, which suggests that the aqueous Shilajit preparation was less effective [13]. Similarly, Alqarni et al. found that after 24 hours, IC₅₀ values in KB-1 oral cancer cells were roughly 1.05 mg/mL, slightly decreasing with longer exposure [7]. Normal human gingival fibroblasts, on the other hand, showed IC₅₀ values of 4-5 mg/mL, indicating that Shilajit remained selectively more toxic to cancer cells even at higher effective doses.

This is supported by wider screening by Phaechamud et al., who reported ED₅₀ (effective dose equivalent to IC₅₀) values of 89 µg/mL (A549 lung), 96 µg/mL (HepG2 liver), 225 µg/mL (MDA-MB-231 breast), and 286 µg/mL (SKOV-3 ovarian) cells, indicating that mineral pitch may potentially be much more effective depending on the specific cancer type and formulation [17].

Overall, these data suggest that breast cancer cells, especially the most aggressive subtypes, are generally more sensitive than lung or oral cancer cells, possibly a function of their differing redox status, mechanisms of uptake, or baseline antioxidant defences [21-27].

Selective Toxicity Toward Cancer Cells

Perhaps the most consistent and relevant finding throughout was that of preferential toxicity by Shilajit toward malignant cells, with comparative minimal effects on their normal counterparts. Kloskowski et al. showed that treatment with Mumio resulted in significantly reduced viability of T24 and 5637 urinary bladder cancer cells, while the cytotoxicity toward normal SV-HUC-1 uroepithelial cells was substantially lower [9]. A similar selectivity was recorded in breast [16] and oral cancer [7] models, with normal mammary epithelial cells and gingival fibroblasts exhibiting IC₅₀ values markedly higher compared to their malignant counterparts. Such a selective susceptibility argues for a mechanism of Shilajit action that is based on the exploitation of an intrinsic vulnerability peculiar to cancer cells, such as altered metabolism, redox imbalance, or dependency on a particular survival pathway, rather than on nonspecific cytotoxicity [28-31].

Mechanistic Pathways Underlying Anticancer Activity

The cumulative data from the included studies indicate that Shilajit possesses anticancer properties through a number of converging yet distinct molecular mechanisms, generally summarized under four primary pathways: ROS-mediated apoptosis, inhibition of NF-κ B/IKK signaling, suppression of EMT and metastasis, and cell cycle arrest, while one study [11] especially pointed out chemo-potentiation and in vivo organ protection.

ROS-mediated apoptosis and miRNA regulation: In hepatic cancer, Pant et al. presented convincing evidence that mineral pitch induces reactive oxygen species (ROS) and nitric oxide (NO) production, leading to mitochondrial damage, lipid peroxidation, and caspase-dependent apoptosis in Huh-7 cells [15]. Importantly, pharmacological neutralization of ROS partially reversed apoptosis and inhibition of proliferation, confirming oxidative stress as a causal driver rather than a secondary effect. This study further demonstrated modulation of miRNAs, with upregulation of tumor-suppressive miRNA-22 and downregulation of oncogenic miRNA-21, resulting in suppression of c-Myc-driven proliferation. Thus, the ROS-miRNA axis works on two fronts: it attacks cells directly and also tweaks gene expression after transcription [32-34].

Jafari et al. [13] and Alshubaily [14] both found something interesting in their antioxidant studies. Shilajit showed its moderate 2,2-diphenyl-1-picrylhydrazyl (DPPH) scavenging activity at relatively high concentrations, while cytotoxic effects occurred at much lower doses. This apparent paradox underlines the context-dependent redox behaviour of Shilajit, i.e., antioxidant in normal physiological settings but pro-oxidant in malignant cells [35-37]. More researchers in anticancer pharmacology are starting to recognize this dual nature.

NF-κ B/IKK Pathway Inhibition: According to Kordestani et al., the primary mechanism identified in breast cancer was the suppression of the IKK/NF-κ B signaling pathway [8]. Shilajit significantly downregulated the mRNA expressions of IKK α, IKK β, p50, and RelB at an IC₅₀ concentration of 280 µg/mL, and this decrease was associated with increased levels of apoptosis as measured using Annexin-V/PI staining techniques. Since NF-κ B acts as a key factor for promoting tumours via inflammation, helping tumours evade immune attack, and resisting chemotherapy/radiation treatment [38-42], this mechanism places Shilajit as a potential anti-inflammatory and survival pathway modulator in hormone-responsive breast cancer.

EMT and metastasis suppression: Rehmani Barouji et al. demonstrated that Iranian mummy strongly inhibited EMT in both MCF-7 and MDA-MB-231 breast cancer-derived cell lines [16]. This was evidenced by the downregulation of components associated with the EMT phenotype, including transforming growth factor β (TGF-β) 1, TGF-β receptor 1 (TGF-β R 1), TWIST 1, NOTCH 1, β-catenin (CTNNB 1), SRC, and vimentin, in conjunction with the upregulation of epithelial cadherin (E-cadherin), thus reducing cell invasive behaviour. These effects were more pronounced in the aggressive MDA-MB-231 line, pointing to the potential relevance in metastatic diseases. Additionally, expanding the implications of these findings beyond breast cancer, Alqarni et al. [7] demonstrated that Shilajit reduces the migration potential of human oral cancer cell lines by targeting both the urokinase-type plasminogen activator (uPA) and its receptor (uPAR) as well as inhibiting key chemokine signalling pathways that govern chemotactic behaviour and extracellular matrix degradation [43-45]. Collectively, these studies suggest Shilajit is a promising candidate for development as an anti-metastatic agent in epithelial malignancies.

Cell cycle arrest: Kloskowski et al. described the effects of Mumio in urinary bladder cancer [9]. They showed that Mumio was capable of inducing cell cycle arrest at the G0/G1 or S phase, followed by apoptosis, with significantly higher effects in cancer cells than in normal urothelial cells. This inhibition of the cell cycle provides one more mechanism of Shilajit for the suppression of tumor growth while maintaining selectivity. The cell cycle arrest is inherently selective because normal cells retain functional G1/S checkpoints (via intact p53/Rb pathways) while cancer cells have defective checkpoints and continue proliferating or undergo apoptosis [46-49].

Chemo-potentiation and organ protection in vivo: Jambi and Abdulaziz Alshubaily showed that Shilajit enhances the effectiveness of the conventional chemotherapy with cyclophosphamide, methotrexate, and 5-fluorouracil on osteosarcoma in lab rats while decreasing the liver and kidney damage caused by the spread of cancer (metastases) [11]. Indeed, in rats treated with Shilajit, serum markers such as aspartate aminotransferase (AST), alanine aminotransferase (ALT), alkaline phosphatase (ALP), creatinine, urea, and uric acid were significantly normalized along with the restoration of histopathological architecture. Although specific molecular targets were not clearly defined, these results indicate that Shilajit may act as an adjuvant that increases efficacy while decreasing the systemic toxicity, like other experimentally proven herbal extracts and isolated herbal compounds (e.g., curcumin, resveratrol, and matairesinol) [50-52].

In fact, these mechanisms are not mutually exclusive but represent a multitarget pleiotropic action where Shilajit induces apoptosis, blocks survival pathways, suppresses metastasis, and protects normal tissues, placing it as a potential candidate for further investigation as an evidence-based anticancer agent.

Limitations and Translational Outlook

Despite these promising results, there are several limitations that need to be considered. First, all the included studies were preclinical; therefore, controlled human trials are lacking, and this fact does not allow direct clinical translation. Second, there was high heterogeneity in Shilajit source, extraction methods, concentrations, and experimental endpoints, reflecting the absence of standardized formulations. Since the chemical composition of Shilajit depends on the geographic origin and further processing, reproducibility and standardization of the dosage continue to constitute one of the most serious challenges. Thirdly, an additional key limitation is the lack of pharmacokinetic and bioavailability data, which critically limits translational relevance. Fourth, the moderate risk of bias in the in vivo study [11] and Category 3 reliability ratings in in vitro studies [7-9,13-17] call for cautious interpretation. Unblinded histopathological assessment might have spuriously inflated estimates of tissue protection. Yet, consistency across multiple studies strengthens confidence in Shilajit bioactivity despite methodological limitations. Results therefore support only preliminary anticancer potential that is suitable for weight-of-evidence assessment and hypothesis generation; any definitive efficacy claims would need to be demonstrated through replication in rigorously designed, blinded studies. Thus, findings must be interpreted as provisional evidence pending higher-quality confirmatory research. Lastly, the limitation of the inclusion of only three major databases due to the unavailability of resources increases the risk of incomplete literature capture.

However, there is a strong need for additional research due to the consistency of anticancer effects across independent studies, cancer models, and mechanistic pathways. Standardizing Shilajit preparations, carrying out thorough pharmacological and toxicological investigations, and moving forward with carefully planned clinical trials should be the main goals of future research, especially when it comes to adjunctive therapy.

## Conclusions

Shilajit demonstrates multimodal anticancer-related activities in experimental models, according to the available data. The results of this systematic review make Shilajit a strong contender for additional translational and integrative oncology research, even though clinical validation is still needed. Future research should concentrate on the characterization of dose-response and the explanation of the mechanisms involved in the synergistic interactions with the conventional chemotherapies in order to increase the therapeutic efficacy, particularly because of its ability to lessen the toxicity caused by the treatment while promoting the cancer-killing effect.
